# Supplementary material for: Characterisation and localisation of plant metabolites involved in pharmacophagy in the turnip sawfly
Source: PLoS One. 2023 Oct 5;18(10):e0291180. doi: 10.1371/journal.pone.0291180 (PMC10553352; doi:10.1371/journal.pone.0291180)
Supplement: Attachment — Submitted filename: Response_to_Reviewers_Brueggemann_et_al_R1_14_08_2023.docx [file pone.0291180.s001.docx]

Manuscript Number: PONE-D-23-18631

Title: Characterisation and localisation of plant metabolites involved in pharmacophagy in the turnip sawfly

Dear Dr. Oliveira,

Thank you very much for giving us the opportunity to revise our manuscript and thanks to all reviewers for their helpful comments. We carefully revised our manuscript following the suggestions by the reviewers and highlighted all changes.

Please find below the comments by the handling editor and reviewers and our replies to each comment.

Kind regards,

Caroline Müller

**GENERAL CHANGES**

All references in the text were formatted within the sentences. instead of after the sentences. The reference for Metabolights was added (lines 175-176) and the ethics statement was moved up (lines 183-185). The following corrections in the figures were made:

Fig. 2: The rightmost structure was missing one negative load at the lowest CH_2_ group, which was added now.

Fig. 3: As correct stated by reviewer#2, the fragments in the middle section had redundant carbons and were removed.

Fig. 4: The label of the x-axis was changed to lower case letters.

Fig. 7: The label of the x-axis was changed to lower case letters.

All figures were checked by the PACE browser software.

**COMMENTS BY REVIEWER #1 AND REPLIES**

REVIEWER #1:
1. different timepoints of sample collection for metabolomic analysis can be done in the future experiment.

REPLY: Thank you very much for the inspiring ideas for future projects.

Different time points were sampled in the past, but did not really show a clear pattern. We would like to try to disentangle the effects in the future.

2. the potential function of focal metabolites will be very interesting to explore through bioassay.

REPLY: Yes, indeed, there may be even more effects than impacts on mating behaviour and defence. However, for bioassays these compounds would need to be purified, as they are not commercially available. We added a part about possible future bioassays in the manuscript in lines 218-221.

3. a transcriptional comparison between C+ and C- adults may be helpful for mining the potential gene involved in the compound modification process.

REPLY: In a previous study we investigated indeed differences in transcriptional pattern between C+ and C- adults, which can be found in Paul SC, Dennis AB, Tewes LJ, Friedrichs J, Müller C (2021) Consequences of pharmacophagous uptake from plants and conspecifics in a sawfly elucidated using chemical and molecular techniques. bioRxiv

<https://doi.org/10.1101/2021.02.09.430406>. As so little is known about the metabolism of these compounds, no clear suggestions about underlying genes can be made from this dataset yet.

**COMMENTS BY REVIEWER #2 AND REPLIES**

REVIEWER #2: The article is well written, but I recommend some adjustments for acceptance:

1. I recommend reformulating the last paragraph of the introduction (lines 72 to 87). The way it was written, it fits better in materials and methods. I suggest a shorter paragraph presenting the research objectives.

REPLY: Many thanks for your kind words and your helpful suggestions. We shortened the last paragraph of the introduction, highlighting the research objectives (now lines 72-84). Some parts were moved to the material and methods section (now lines 106ff).

2. All molecules eliminated in figure 3 have an extra carbon at the end of the broken bond. Fix it.

REPLY: Thank you for this important comment. We corrected the respective figure.
